# Le fibromatosis colli: une tumeur cervicale rare du nourrisson

**DOI:** 10.11604/pamj.2020.37.370.24635

**Published:** 2020-12-23

**Authors:** Nogognan Ignace Lengane, Milckisédek Judicaël Marouruana Some, Mohamed Tall, Alain Saga Ouermi, John Pepin Nikiema, Joséphine Wendemy Ouoba, Moussa Kadyogo, Moustapha Sereme

**Affiliations:** 1 Service ORL et Chirurgie Cervicofaciale, CHU Régional de Ouahigouya, Ouahigouya, Burkina Faso,; 2 Service d´Imagerie Médicale, CHU Régional de Ouahigouya, Ouahigouya, Burkina Faso,; 3 Service de Pédiatrie, CHU Régional de Ouahigouya, Ouahigouya, Burkina Faso,; 4 Service ORL et Chirurgie Cervicofaciale, CHU Pédiatrique Charles De Gaulle, Ouagadougou, Burkina Faso,; 5 Service ORL et Chirurgie Cervicofaciale, CHU Bogodogo, Ouagadougou, Burkina Faso

**Keywords:** Fibromatosis colli, muscle sternocléidomastoïdien, torticolis, nourrisson, Fibromatosis colli, sternocleidomastoid muscle, torticollis, infant

## Abstract

Le fibromatosis colli ou pseudotumeur infantile du muscle sternocléidomastoïdien est une cause rare de masse cervicale bénigne du nouveau-né et du nourrisson. L´étude a concerné tous les patients admis pour une tuméfaction cervicale et chez qui le diagnostic de fibromatosis colli a été posé de mars 2016 à février 2020. Cinq patients ont été retenus. La tuméfaction cervicale est apparue au cours du premier mois de vie chez tous nos patients. Le diagnostic de fibromatosis colli a été posé à l´échographie. Tous les patients ont bénéficié d´un traitement conservateur. Le fibromatosis colli est une cause relativement rare de masse cervicale du nouveau-né et du nourrisson.

## Abstract (en)

Fibratosis colli, or infantile pseudotumor of the sternocleidomastoid muscle, is a rare cause of benign cervical mass in newborns and infants. This study involved all patients admitted with cervical swelling and diagnosed with colli fibromatosis from March 2016 to February 2020. Five patients were retained. In all patients cervical swelling occurred in the first month of life. No patient had had obstetric trauma. The diagnosis of fibromatosis colli was based on ultrasound. All patients received medical treatment. Fibratosis colli is a relatively rare cause of cervical mass in newborns and infants.

## Introduction

Le fibromatosis colli ou pseudotumeur infantile du muscle sternocléidomastoïdien est une cause rare de masse cervicale bénigne du nouveau-né et du nourrisson. Il est rencontré dans 0,4% des naissances vivantes. Il se présente sous la forme d´une masse ferme, indolore au sein du muscle sternocléidomastoïdien [1,2]. Nous rapportons une série de 5 cas de fibromatosis colli, avec une revue des aspects diagnostiques, thérapeutiques et évolutifs.

## Méthodes

L´étude a concerné tous les patients admis pour une tuméfaction cervicale et chez qui le diagnostic de fibromatosis colli a été posé de mars 2016 à février 2020. Cinq patients ont été retenus. Les informations sur le mode d´accouchement, les signes cliniques, l´imagerie, le traitement, et l´évolution ont été colligées. Tous les patients ont bénéficié d´un examen clinique et d´une échographie. Les patients étaient suivis tous les 2 mois.

## Résultats

**Figure 1:**
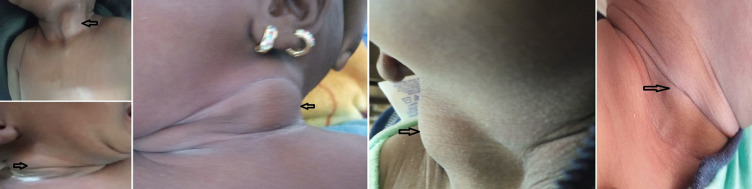
tuméfaction cervicale (flèche)

**Figure 2:**
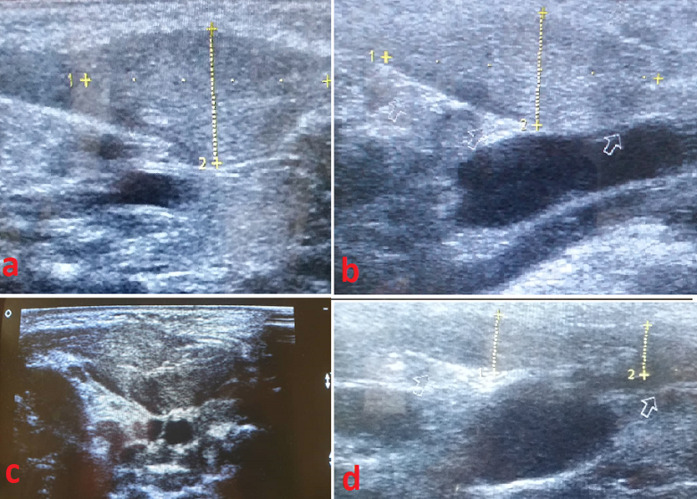
coupes échographiques longitudinales montrant un épaississement du muscle sternocléidomastoïdien (a, b, c); comparé à un coté normal (d)

Durant la période d´étude, 5 patients ont été admis pour une tuméfaction cervicale (Figure 1). Elle est apparue au cours du premier mois de vie chez tous nos patients. Quatre patients étaient de sexe masculin. L´accouchement était à terme par voie basse chez tous nos patients. Il n´y avait pas de notion de traumatisme obstétrical. La tuméfaction siégeait à droite chez 2 patients. Un torticolis était retrouvé chez 3 patients (Tableau 1). Le diagnostic de fibromatosis colli a été posé à l´échographie chez tous nos patients (Figure 2). Aucune exploration paraclinique supplémentaire n´a été effectuée. Tous les patients ont bénéficié d´un traitement conservateur consistant en une rotation de la tête du côté de la lésion lorsque l´enfant est au dos et au cours du sommeil. L´évolution a été favorable chez tous nos patients dans un délai moyen de 7 mois.

**Tableau 1:** caractéristiques des patients

No.	Sexe	Age (semaines)	Coté	Traumatisme obstétrical	Torticolis
**1**	F	8	Gauche	Non	Oui
**2**	M	4	Droit	Non	Oui
**3**	M	6	Gauche	Non	Non
**4**	M	5	Droit	Non	Oui
**5**	M	4	Droit	Non	Non

## Discussion

Le fibromatosis colli est une prolifération fibroblastique bénigne du muscle sternocléidomastoïdien. Bien que l´étiologie soit inconnue, il semble être lié à une ischémie du muscle en rapport avec un traumatisme obstétrical. Une notion de traumatisme obstétrical est retrouvée dans plus de 50% des cas [1,3]. Sa prévalence est d´environ 0,4% des naissances vivantes [2,3]. Il siège plus fréquemment à droite dans 75% des cas avec une prédominance masculine, comme retrouvée dans notre série [2-4]. Il est une des causes de torticolis néonatal. Le torticolis est présent dans environ 20% des cas. Une atteinte bilatérale est rarement décrite [1]. Il se présente comme une tuméfaction cervicale ferme, mobile sous la peau faisant corps avec le muscle sternocléidomastoïdien. Il apparait entre 2 à 4 semaines après la naissance, en général suite à un accouchement difficile (extraction par ventouse ou forceps) [1,5].

L´échographie cervicale est la technique d´imagerie de choix. Elle est accessible, non invasive, fiable avec une sensibilité de 100% rapportée dans la littérature. Elle objective une tuméfaction fusiforme de 2 à 3 cm siégeant dans les deux-tiers inférieurs du muscle, et dont les mouvements sont synchrones avec le muscle sternocléidomastoïdien [1,3-6]. A la tomodensitométrie, le muscle apparait élargi, isodense. A l´imagerie par résonnance magnétique, il y´ a une diminution du signal de la masse en T2 comparé au signal en T1, liée à la présence de tissu fibreux. L´étendue de l´atteinte musculaire est mieux appréciée par l´imagerie par résonnance magnétique que par l´échographie [1]. La cytoponction est indiqué pour confirmer le diagnostic et éliminer d´autres causes congénitales, inflammatoires et tumorales. Elle permet d´éviter des biopsies ou des chirurgies non nécessaires. Elle met en évidence une prolifération fibroblastique, une atrophie musculaire, des cellules musculaires géantes et une absence de cellules inflammatoires. Le diagnostic différentiel comporte le kyste branchial, les adénopathies, le rabdomyosarcome et le neuroblastome [1-3].

Le traitement est conservateur avec une physiothérapie avec des exercices d´étirement cervicale [1]. Lee *et al*. ont proposé un protocole de physiothérapie consistant en 3 séries de 15 étirements manuels du cou pendant 1 seconde et une pause de 10 secondes entre chaque étirement. Cet exercice est effectué 3 fois par semaine. Les parents doivent également tourner la tête de l´enfant du côté de la lésion lorsqu´ils le portent au dos ou durant son sommeil. La physiothérapie est efficace dans 80 à 90% des cas. Quand elle est débutée dans les 4 premiers mois, la guérison est obtenue en 3 à 4 mois. Une guérison spontanée peut également survenir [2,5,7,8]. Lin notait une réduction du volume de la fibrose de 83,6% à 2 mois à 59,9% à 9 mois et une réduction supplémentaire jusqu´à 40% à partir de 1an [9]. La chirurgie est recommandée en cas de persistance des symptômes au-delà d’un an. Il s´agira d´une ténotomie ouverte ou d´une exérèse de la masse [2,7,10]. Récemment l´utilisation de la toxine botulique en injection intra lésionnelle est explorée comme une alternative à la chirurgie [4].

## Conclusion

Le fibromatosis colli est une cause relativement rare de masse cervicale du nouveau-né et du nourrisson. Les caractéristiques radiologiques permettent de le différencier des autres masses cervicales de l´enfant.

### Etat des connaissances sur le sujet

- L´imagerie médicale occupe une place importante dans le diagnostic. Le traitement est en général conservateur.

### Contribution de notre étude à la connaissance

- Le fibromatosis colli a régressé complètement après une physiothérapie passive. Aucune notion de traumatisme obstétrical n´a été retrouvée comme facteur étiologique. L´échographie a été la technique d´imagerie de choix pour le diagnostic dans notre contexte.
